# Relationships between Agronomic Traits of Moringa Accessions and In Vitro Gas Production Characteristics of a Test Feed Incubated with or without Moringa Plant Leaf Extracts

**DOI:** 10.3390/plants11212901

**Published:** 2022-10-28

**Authors:** Addisu Endalew Zeru, Abubeker Hassen, Zeno Apostolides, Julius Tjelele

**Affiliations:** 1 Department of Animal Science, University of Pretoria, Pretoria 0002, South Africa; 2 Department of Biochemistry, Genetics and Microbiology, University of Pretoria, Pretoria 0002, South Africa; 3 Range and Forage Sciences, Agricultural Research Council, Pretoria 0002, South Africa

**Keywords:** methane mitigation, organic matter digestibility, ruminants, total flavonoids, total gas, total phenolics

## Abstract

The use of medicinal plants and their extracts has recently attracted the attention of many researchers as a methane (CH_4_) mitigation strategy. This study evaluated the relationship of agronomic traits of Moringa accessions with in vitro gas production measurements and feed digestibility from ruminants. Twelve Moringa accessions were grown at the Roodeplaat experimental site of the Agricultural Research Council in Pretoria, South Africa. Agronomic traits, such as seedling survival rate, leaf yield, canopy and stem diameter, plant height, number of primary branches, plant vigor, greenness, chlorosis, disease and pest incidences were recorded. The leaves were harvested in the fifth month after transplanting to the field. Freeze-dried leaves were extracted with methanol, and their total phenolic and total flavonoid contents were determined. The extract was applied at a dose of 50 mg/kg of dry matter (DM) feed for in vitro gas production studies. Most of the growth and agronomic traits, i.e., seedling survival rate, leaf yield, canopy diameter, plant height, number of primary branches, the score of plant vigor, and greenness, total phenolics and flavonoids were significantly different among the accessions except for stem diameter and chlorosis score. All accession leaf extracts significantly reduced the total gas and CH_4_ production compared with the control with equal or higher in vitro organic matter digestibility. Higher CH_4_ inhibition was obtained in *Moringa oleifera* (*M. oleifera*) A3 (28.4%) and A11 (29.1%), whereas a lower inhibition was recorded in A1 (17.9%) and A2 (18.2%). The total phenolic (0.62) and total flavonoid (0.71) contents as well as most agronomic traits of the accessions were positively correlated with the CH_4_ inhibition potential of the accessions. *Moringa oleifera* accessions A3, A8 and A11 resulted in higher in vitro CH_4_ inhibition potential and improved organic matter digestibility of the feed with equal or higher adaptability performances in the field. Thus, there is a possibility of selecting Moringa accessions for higher antimethanogenic activity without compromising the feed digestibility by selecting for higher total phenolics, total flavonoids and agronomic performances traits. There is a need for further study to determine the long-term adaptability of promising accessions in the study area with concurrent antimethanogenesis efficacy when used in the diet of ruminant animals.

## 1. Introduction

Ruminant animal production has been among the most demanded sectors of livestock and has a pivotal role in sustainable food supply and economic benefits [1,2]. However, CH_4_ produced in ruminants as a byproduct of anaerobic microbial fermentation in the rumen has been increasingly a shadow to production [3,4]. Methane emitted from ruminants not only has a global warming effect, but also results in energy loss and reduces the productivity of animals [5]. Thus, the development and implementation of applicable strategies to reduce enteric CH_4_ production and improves the dietary energy use efficacy of animals are urgently required [6]. Hence, the use of medicinal plants and their extracts has recently attracted the attention of many researchers as a mitigation strategy. This is because of its safety to produce organic animal products, suitability and relatively economical alternative feed additives in the ruminant feeding system to reduce enteric CH_4_. In this regard, the multipurpose functions of *M. oleifera* as antioxidants, anticancerous, antidiabetic, anti-inflammatory agents, antimicrobial properties and nutritional benefits have increased its demand and have been among the most intensively cultivated medicinal plants [7,8].

Plant survival, growth and development performances were different among and within the Moringa species [9,10]. However, plant growth, adaptability and bioactivities interact with multiple factors [11,12]. Hence, the genetic variability that existed among and within the Moringa species was implicated as a reason for the variation in plant growth, leaf yield and agro-morphological characteristics (leaflet size and shape, branching habit, time to flowering, pod width and pod length of the plants), and played a remarkable role for its application in ruminant production [9,10]. Similarly, the distribution patterns of bioactive plant secondary metabolites (PSMs) are also different among genetics and plant parts, such as flowers, seeds, stems, leaves and roots, which are tissue- or organ-specific [13,14]. The leaves of three Moringa species (*M. oleifera*, *M. stenopetala. M. peregrina*) grown in Egypt showed different total phonics (4870–10,870 mg GAE/100 g), total flavonoids (3120–6280 mg QE/100 g) and various phenolic compounds concentrations [15]. *Moringa oleifera* accessions grown in Algeria, Chad and Haiti differed in their total polyphenols (2545–3552 mg/100 g), salicylic acid (0.14–0.33 mg/100 g) and ferulic acid (6.61–9.69 mg/100 g) contents [16]. The authors linked the variations to genotype and environment interactions. A study conducted on three varieties of *Labisa pumila* Benth leaf extracts showed different antioxidant activities [17]. Similarly, Skrovankova [12] and Skrovankova [11] also revealed that genotype significantly affected the bioactive metabolites in berries and green coffee, respectively. The biosynthesis processes of PSMs are strictly controlled by genes [14,18], which subsequently influence the antimethanogenic potentials of the plants. Accessions of Moringa species may have different adaptability and growth performances, which differently affect the gas production characteristics of feeds when their extracts are used as an additive in ruminants.

The gas production and antimethanogenic potential of the Moringa accession extracts may also be associated with their agronomic and growth performances. One of the studies stated that an increased accumulation of PSMs with higher biomass, stem diameter, root diameter and plant height in woody and perennial medicinal plants [14]. This may result in better antimethanogenic potential during their application in rumen modulation. Hence, determining the optimal harvesting stage may help to obtain the highest metabolite composition, yield of PSMs and higher bioactivity, including the antimethanogenic activity specific to Moringa plants. Phenolics and flavonoids are highly abundant in Moringa and showed different bioactivities (i.e., antioxidant, antimicrobial, anticancerous, antidiabetic, antilipogenic and immunomodulatory activities) [19]. Several studies also reported phenolic and flavonoid compounds as the main active components against bacteria, protozoa and fungi [20,21,22]. Accompanied with the mentioned studies, strong antimicrobial activities were obtained from metabolites having functional groups of phenolic acids, phenolics and terpenoids [23,24,25,26], which thereby affect the antimethanogenic potential of the extracts in different ways. The inclusion of some commercial flavonoids (quercetin, flavone, myricetin, rutin, kaempferol, and naringin) also exhibited 5–9 mL/g DM CH_4_ inhibition, despite most of them negatively affected the feed digestibility except for quercetin and naringin [27]. Some phenolic acids (i.e., caffeic acid, *p*-coumaric acid, trans-cinnamic acid, and catechin hydrate acids) also inhibited CH_4_ production with no significant effect on the total gas, VFAs profile and pH [28]. These imply that the antimethanogenic potentials of Moringa can be correlated with total phenolics and total flavonoid contents. However, the relationship between gas production characteristics and antimethanogenic potentials with its agronomic traits and the contents of total phenolics and total flavonoids has been poorly understood. Thus, generating such information for Moringa is crucial to identify more productive varieties of Moringa accession for specific areas and increases its antimethanogenic efficacy with a co-benefit of improving feed digestibility. 

Thus, the current study was conducted mainly to investigate if the agronomic traits, total phenolic and total flavonoid contents of Moringa accessions could be different when they grew in the same environment due to genetic variation, which in turn affects the in vitro fermentation characteristics of ruminant feed. This increases our understanding of many influencing factors to develop a consistent Moringa leaf extract product effectively used for the mitigation of CH_4_ from ruminants. However, to the best of our knowledge, variations in plant growth and agronomic traits, total phenolics and total flavonoids have not been associated with the gas production and antimethanogenic potential of Moringa accessions in previous studies. Therefore, this study intended to evaluate the relationship between the agronomic traits (seedling survival rate, leaf yield, canopy and stem diameter, plant height, number of primary branches, the score of plant vigor, greenness and chlorosis as well as disease and pest incidences) and the phytochemical contents (i.e., total phenolics and total flavonoids) of the Moringa accessions grown in the subtropical climate of Pretoria, South Africa, with the in vitro gas production characteristics of *Eragrostis curvula (E. curvula*) hay incubated with an additive of Moringa accession leaf extracts.

## 2. Results

### 2.1. Agronomic Performances of Moringa Accessions during the Establishment Year

The agronomic performances of the Moringa accessions at five months of the establishment year are illustrated in Table 1. The studied accessions significantly (*p* < 0.01) different in their seedling survival rate in the field and ranged from 45% in *M. oleifera* A10 to 78% in A2. The accession (A11) collected from a private farmer in Pretoria, South Africa was expected to show the highest survival rate; however, some accessions collected in different areas of Kenya (A2, A5, A8) and Mali (A3 and A4) exhibited equal or higher seedling survival rates than A11. Whereas *Moringa stenopetala (M. stenopetala*) showed a relatively poor survival rate (59%) compared with most of the *M. oleifera* accessions.

Most of the studied plant agronomic traits, such as the canopy diameter, plant height, number of primary branches as well as fresh and dry leaf yield, of the accessions were significantly *(p* < 0.05) varied except for the stem diameter and chlorosis score. In this stage of growth, *M. oleifera* A3 (188 cm), A2 (164 cm), A4 (160 cm) and A5 (166 cm) were higher than other accessions, whereas A10 (105 cm) from *M. oleifera* and *M. stenopetala* (47 cm) were the shortest among the accessions in plant height (Table 1). *Moringa oleifera* A1, A2, A3, A5 and A8 exhibited significantly higher leaf yields than the other accessions. In terms of the amount of fresh leaf yield, the highest yield was obtained from A2 (2981 kg/ha, 828 kg/ha), followed by A5 (2149 kg/ha), A3 (2144 kg/ha), A8 (2107 kg/ha) and A1 (2063 kg/ha). Similarly, *M. oleifera* A2 (828 kg/ha) showed the highest amount of dry leaf yield, followed by A3 (608 kg/ha), A8 (604 kg/ha), A5 (590 kg/ha) and A1 (553 kg/ha); however, all these accessions statistically had equivalent amounts of leaf yields.

The scored values of plant vigor and greenness were also significantly (*p* < 0.05) different among the accessions (Table 1). *Moringa oleifera* A2, A3, A4, A5, A8 and A11 exhibited significantly higher scored values of plant vigor, which indicates their healthiness and how they look in terms of their overall performance in the field. Whereas the *M. oleifera* A10 and *M. stenopetala* had the lowest plant vigor performances in the field. Most of the accessions that showed superior plant vigor performance also had higher greenness values; however, some accessions, such as A9 and A10, which scored lower on plant vigor, exhibited better greenness values. The problem of chlorosis was also noted in most of the accessions starting from the fourth month of transplanting and gradually increased from the bottom (older leaves) to upwards (young leaves), although severity did not vary significantly (*p* > 0.05). However, identifying the cause of the chlorosis problem was not within the scope of the current study and thus not known though aging of the leaves might likely be the cause for the problem. Hence, optimizing the leaf harvesting age of Moringa plant growth might be essential to obtain quality leaves with increased leaf production. In this study, disease and pests were also assessed regularly; however, no incidence was observed within five months of the experimental period. Generally, *M. oleifera* A2 and A3 seemed to be superior in most of the agronomic traits, whereas *M. oleifera* A6 and A10 and *M. stenopetala* showed inferior performances in most of the parameters (Table 1).

**Figure 1:**
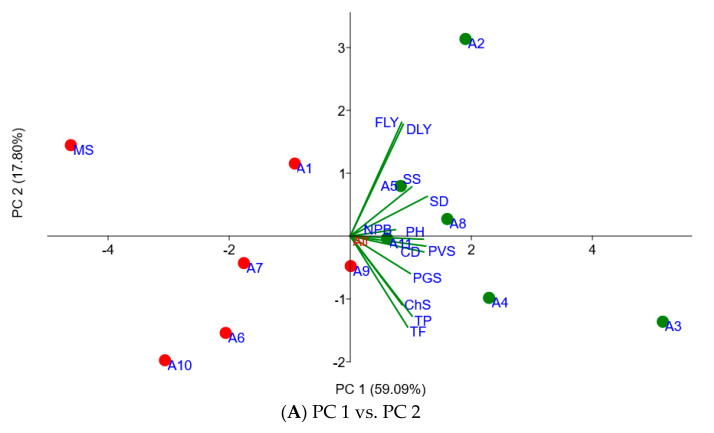

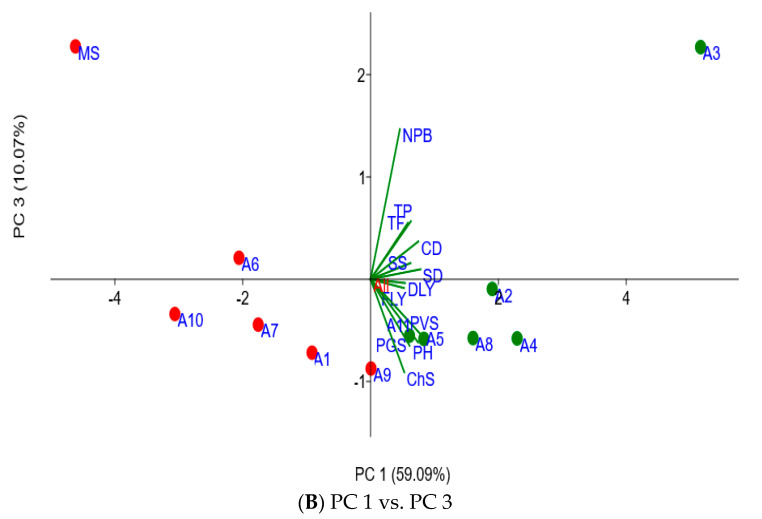
Principal component analysis scatter plots of the Moringa accessions and biplot of agronomic traits at five months of the establishment year. (**A**) refers to the principal component analysis results using the principal component 1 and principal component 2, whereas (**B**) is principal component analysis results using the principal component 1 and principal component 3. CD: canopy diameter; PH: plant height; NPB: number of primary branches; SD: stem diameter; FLY: fresh leaf yield; DLY: dry leaf yield; PVS: plant vigor score; PGS: plant greenness score; ChS: plant chlorosis score; TP: total phenolics; TF: total flavonoids; the green and red dots can be used to differentiate the accessions to higher and lower agronomic traits in their field performances, respectively.

The principal component loadings of the agronomic traits and scores of the accessions obtained from the PCA are presented in Table 2 and Figure 1. The first three major PCs explained 87% of the total variation among the accessions. PCA clarified the variation in accessions in terms of their agronomic performances in the field. Thus, PC 1 alone accounted for 59% of the total variation and was positively correlated with all agronomic traits of the study. However, the plant height, stem diameter, canopy diameter and score of plant vigor were the major parameters responsible for PC 1. PC 2 and PC 3 were responsible for 18% and 10% of the total variation in accessions, respectively. Most of the parameters were negatively correlated with PC 2, although the dry leaf yield, fresh leaf yield, number of primary branches, stem diameter, and seedling survival correlated positively. The fresh leaf yield (51%), dry leaf yield (50%) and total flavonoid content (−40%) were the major parameters responsible for PC 2. Figure 1 also illustrated the scatter dot plots of the accessions and biplot distribution of the agronomic parameters recorded at five months of the establishment year. Thus, the score plot distribution of the accessions seemed to divide them into two main groups, as referenced to PC 1 with 59% variation. The vectors of all agronomic traits as well as total phenolic and total flavonoid contents are laid in the same direction with the accessions represented with green dots while opposite to the accessions denoted by red dots. Hence, accessions marked with green dots (A2, A3, A4, A5, A8 and A11) showed higher adaptability in most of the studied agronomic traits, total phenolic and total flavonoid contents than those accessions marked with red dots.

### 2.2. In Vitro Total Gas, Methane Production and Organic Matter Digestibility

As presented in Table 3, the total gas production (TGP) from anaerobically incubated *E. curvula* hay treated with leaf extracts of all studied Moringa accessions was significantly (*p* < 0.01) different from 160.0 mL/g DM to 172.6 mL/g DM. However, this result was lower when compared with the total gas produced from the control (203.8 mL/g DM). The feed fermentation kinetics were not affected negatively by the extracts used in this study, which was proven with their equal or higher IVOMD compared with the control. Consistently, the total CH_4_ produced from the substrate feed treated with the studied accession extracts also varied from 4.52 mL/g DM in *M. oleifera* A11 to 5.23 mL/g DM in A1. This result was significantly lower compared with the CH_4_ released from the control (6.33 mL/g DM). The CH_4_/IVOMD of the substrate feed and their percentage of antimethanogenic potential were also substantially different among the accessions. Hence, the percentage of CH_4_ inhibition recorded among the accessions ranged between 18% (A1) and 29% (A11). Statistically, the highest CH_4_ inhibition potential was obtained in A11 (29%) and A3 (28%), whereas the lowest CH_4_ inhibition was recorded in A1 (18%) and A2 (18%) among the accessions.

**Figure 2:**
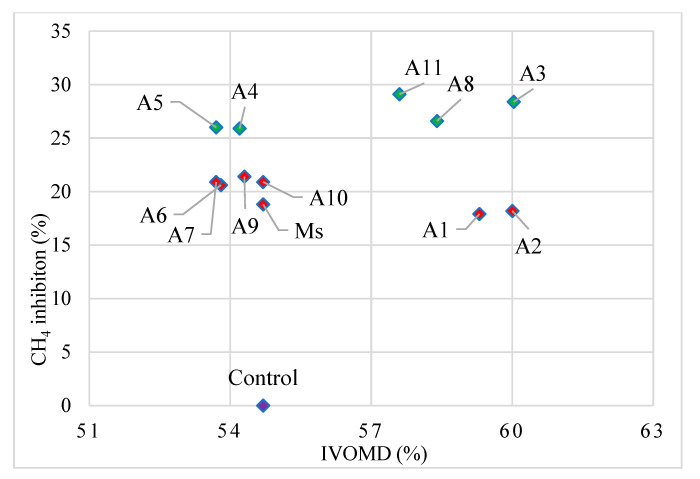
Scatter diagram of methane inhibition vs. in vitro organic matter digestibility of *Eragrostis curvula* hay fermented with the leaf extracts of Moringa accessions. IVOMD: in vitro organic matter digestibility; green and red diamonds showed higher and lower methane inhibitor accessions, respectively.

The IVOMD of the *E. curvula* hay fermented with extracts of the studied Moringa accession was considerably different (*p* < 0.01) and ranged from 54% (A5) to 60% (A3) (Table 3). The IVOMD obtained from the *E. curvula* hay fermented with extracts of the studied accessions was higher than or equal to that of the control (55%). Based on their effect on OM digestibility, the studied accessions seemed to be divided into two distinct groups, as indicated in Figure 2. Thus, the five *M. oleifera* accession extracts of A1 (59%), A2 (60%), A3 (60%), A8 (59%) and A11 (58%) significantly (*p* < 0.01) improved the IVOMD. The other accessions, such as A4 (54.2%), A5 (53.7%), A6 (53.8%), A7 (53.7%), A9 (54.3%), A10 (54.7%) and *M. stenopetala* (54.7%), showed equal IVOMD with the control, *E. curvula* hay (54.7%) incubated without any plan extract additive. *Moringa oleifera* A1 and A2 established a superior IVOMD than the control; however, their antimethanogenic potentials were lower than that of most of the accessions. On the other hand, A4 and A5 exhibited higher CH_4_ inhibition, but they did not improve the IVOMD of the tested feed. None of the accessions showed a decrease in IVOMD compared with the control, which infers that none of the accession extracts negatively affected the feed fermentation kinetics. Generally; however, five of the studied accessions improved the IVOMD of *E. curvula* hay compared with the control, and only A3, A8 and A11 provided both benefits of higher CH_4_ inhibition potential and improved feed OM digestibility (Figure 2). *Eragrostis curvula* hay treated with *M. stenopetala* extracts exhibited higher total gas and CH_4_ production with lower CH_4_ inhibition potential and the IVOMD recorded for *M. stenopetala* plant extract compared with most *M. oleifera* accession extracts. This shows us the presence of wide genetic variability within the Moringa accessions and between the species, which eventually influences the effect of extracts on their anti-methanogenic potential and feed digestibility.

### 2.3. Total Phenolic and Total Flavonoid Contents

As presented in Table 4, accessions showed significantly *(p* < 0.01) different total phenolic and total flavonoid contents in the study. In terms of magnitude, the highest total phenolic content was obtained in *M. oleifera* A3 (4698 GAE/100 g extract), followed by A8 (3998 GAE/100 g extract), A4 (3927 GAE/100 g extract) and A11 (3800 GAE/100 g extract), while *M. oleifera* A2 (3489 mg GAE/100 g) maintained the lowest total phenolics. The total flavonoid content also significantly varied from 1834 mg QE/100 g extract (A1) to 2915 mg QE/100 g extract (A3). *Moringa oleifera* A3 also showed the highest total flavonoid content, followed by A4 (2391 mg QE/100 g extract), A5 (2311 mg QE/100 g extract), A11 (2275 mg QE/100 g extract) and A8 (2250 mg QE/100 g extract). *Moringa oleifera* A3, A4, A8 and A11 exhibited higher contents of the mentioned phytochemicals in this study. Whereas A1 and A2 of *M. oleifera and M. stenopetala* were inferior in these metabolites. Along with the *M. oleifera* A3, A8 and A11, A4 and A5 also maintained relatively superior phytochemicals of total flavonoid and total phenolic contents and antimethanogenic potential compared to other accessions. However, A4 and A5 did not improve the OM digestibility of the substrate feed. In contrast, A1 and A2 recorded lower total phenolic and total flavonoid contents, while they improved IVOMD compared with the control. This result infers that IVOMD is not only dependent on the total phenolic and total flavonoid contents, but also might be affected by other specific metabolites in a very specific way. However, these phytochemical contents significantly correlated with total gas, CH_4_, CH_4_/IVOMD production and CH_4_ inhibition potentials of the accessions. Hence, the CH_4_ inhibition potential of accessions showed significant positive Pearson correlation coefficients of 0.70699 and 0.62057 with total flavonoid and total phenolic contents, respectively (Table 4).

### 2.4. Associating Agronomic Traits of Moringa Accessions with In Vitro Fermentation Characteristics

The Pearson correlation analysis results for agronomic traits with the in vitro fermentation characteristics of *E. curvula* hay treated with the Moringa accession plant extracts are presented in Table 5. Total gas was negatively and significantly correlated (*p* < 0.05) with most agronomic traits, such as canopy diameter, plant seedling survival, plant height, stem diameter, vigor and plant greenness, but not with leaf yield and number of primary branches of the accessions (Table 5). However, the total CH_4_ produced (negatively) and % CH_4_ inhibition (positively) showed a significant correlation only with the seedling survival rate and leaf greenness score of the plants, and most agronomic traits were significantly and negatively associated with the CH_4_/IVOMD, except for the chlorosis score (*p* < 0.05). The IVOMD of the tested *E. curvula* hay was also positively and significantly correlated with the fresh and dry leaf yield, the number of primary branches, plant height, and stem diameter of the accessions at *p* < 0.05. The total flavonoid and phenolic contents of the accessions were also positively correlated with several agronomic traits, such as canopy diameter, the number of primary branches, plant height, stem diameter, as well as the score of plant vigor and greenness (*p* < 0.05). Hence, this correlation analysis generally showed a positive association of the CH_4_ inhibition potentials of the accessions with higher agronomic performances of the accessions. 

**Figure 3:**
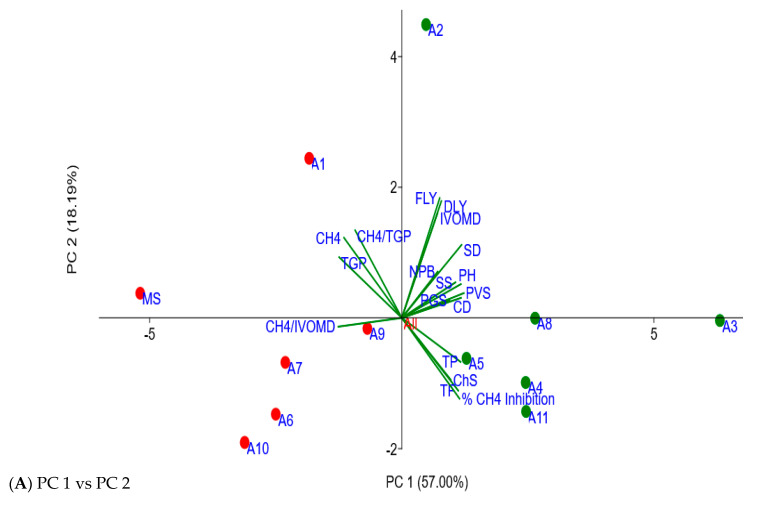

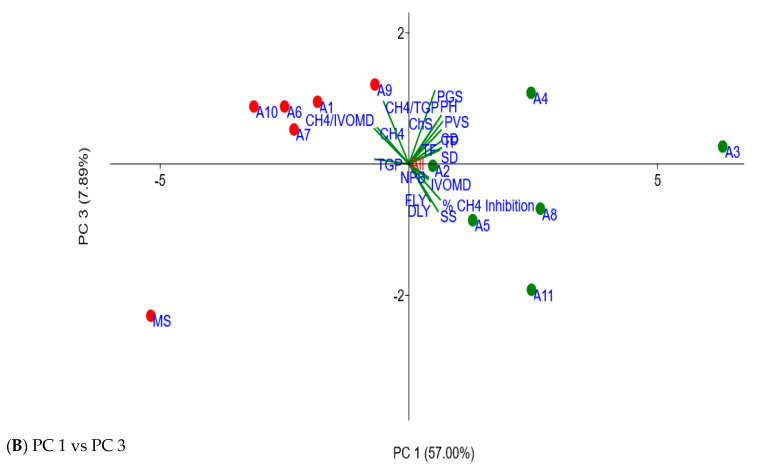
Principal component analysis scatter plots of the studied Moringa accessions, which show the relationship of the different agronomic traits with the in vitro fermentation characteristics. (**A**) refers to the principal component analysis results using the principal component 1 and principal component 2, whereas (**B**) is the principal component analysis results using the principal component 1 and principal component 3. PC: principal component; TGP: total gas production; TP: total phenolics: TF: total flavonoids: IVOMD: in vitro organic matter digestibility. Green and red dots represent the accessions and are used to differentiate them, as the green dots indicate higher methane inhibition and agronomic performances while those denoted by red dots exhibited lower methane inhibition and agronomic performances in the field.

The principal component (PC) loadings and scatter plot of Moringa accessions using their different agronomic traits and in vitro fermentation parameters are presented in Table 6 and Figure 3. Thus, the first three major PCs explained approximately 83% of the total variations of the accessions on the studied parameters. PC 1 alone contributed to 57% of the total variation and was positively correlated with most of the studied parameters. However, it was also negatively correlated with TGP, CH_4_ volume, CH_4_/TGP and CH_4_/IVOMD. Plant height (26%), stem diameter (26%), canopy diameter (26%), plant vigor (27%), TGP (−28%), CH_4_ (−26%), CH_4_/IVOMD (−28%), % CH_4_ inhibition (26%), TP (26%) and TF (27%) contributed better to PC 1. PC 2 and PC 3 were responsible for about 18% and 8% of the total variation obtained in the PCA, respectively. PC 2 was negatively correlated with CH_4_ inhibition, CH_4_/IVOMD, chlorosis score, total phenolic and total flavonoid contents and positively correlated with other parameters of the study. The majority of the variation in PC 2 was attributed to dry leaf yield (40%), fresh leaf yield (41%), IVOMD (36%) and CH_4_/TGP (30%), while PC 3 was highly reliant on CH_4_/TGP (39%), plant greenness (45%), plant height (30%) and seedling survival (29%). As shown in Figure 3, the PCA elucidated the overall relationship of agronomic traits, total phenolic and total flavonoid contents of the accessions with the in vitro fermentation characteristics of *E. curvula* hay treated with their extracts. Thus, the biplots of CH_4_ volume, TGP, CH_4_/TGP and CH_4_/IVOMD were laid in the same direction as the score plot of the accessions, which was characterized by lower CH_4_ inhibition potential (A1, A6, A7, A9, A10, MS). However, these biplots were placed opposite the score plots of accessions (A3, A4, A5, A8 and A11) that showed higher CH_4_ inhibition. The vectors of most agronomic traits, % CH_4_ inhibition, TP and TF contents were laid in the same direction with accessions grouped as higher CH_4_ inhibitors (A3, A4, A5, A8, and A11) as referenced to PC1, which infers the positive relationship of the mentioned parameters. Hence, in this PCA, it is apparent that the higher CH_4_ inhibition potential obtained from A3, A4, A5, A8 and A11 is positively associated with most agronomic traits (plant height, seedling survival, leaf greenness, plant vigor, canopy diameter and stem diameter) as well as the TP and TF contents found in their extracts. These results are accompanied by the results of positive and significant Pearson correlation coefficients of most agronomic traits with the CH_4_ produced per unit of IVOMD illustrated in Table 5. However, only leaf greenness and seedling survival from agronomic traits (Table 5) as well as total phenolic and total flavonoid contents (Table 4) were significantly correlated with the total CH_4_ volume produced. 

**Figure 4:**
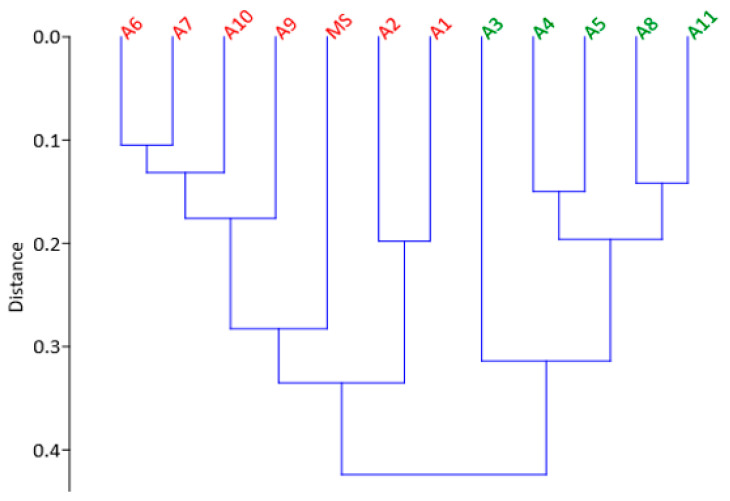
Hierarchical clustering of Moringa accessions using all the in vitro fermentation characteristics and agronomic traits. Similar to the PCA, accessions represented by green showed higher methane inhibition and agronomic performance, while those denoted by red exhibited lower methane inhibition and agronomic performances in the field.

The HCA, illustrated in Figure 4, also showed two main cluster trees and supported the results obtained from the scatter plot distribution of PCA output, presented in Figure 3. *Moringa oleifera* A1, A2, A6, A7, A9, and A10 and MS clustered in one of the main cluster trees, whereas the other accessions of *M. oleifera* (A3, A4, A5, A8 and A11) were grouped on the other main cluster tree of the HCA. This relationship study showed the presence of a positive correlation between most agronomic traits and higher CH_4_ inhibition characteristics of the accessions, in which better adaptable accessions in the field exhibited better inhibition of CH_4_ production. However, *M. oleifera* A2, which showed superior agronomic performance in the field and inferior CH_4_ inhibition potential seemed to be an exception, and this study could not identify and explain the reasons for its variation. 

## 3. Discussion

*Moringa oleifera* accessions and *M. stenopetala* grew in the subtropical climate of Pretoria, South Africa, and exhibited different agronomic performances in the field. The seedling survival rates of *M. oleifera* varied from 45% (A10) to 78% (A2), while *M. stenopetala* showed a 59% survival rate. This rate was lower than the 100% survival reported in *M. oleifera* and 97% in *M. stenopetala* at the Bako Research Center in Ethiopia [29]. However, we could not be warranted to the comparison due to uncertainty about the similarity of environmental conditions of the specified study with the current study. A higher survival rate of 75–100% was also revealed in different *M. oleifera* accessions grown in the tropical climate of the central Philippines [30]. The maximum plant height growth (1.88 m) obtained in *M. oleifera* A3 in the fifth month of growth in the field was lower than the 3 m height attained in the third month in Zimbabwe [23] and the 2 m height recorded at 116 days in the Central Philippines [30]. Moringa accessions were also different in their leaf greenness score in the current study. The variation might be attributed to not only the difference in nitrogen (N) content, but also potassium (K) content [31,32,33] and chlorophyll molecules [34]. Thus, Hou et al. [33] stated that darker green leaves indicated higher N contents, while lighter green leaves showed greater K rates. Similarly, another study conducted on rice also supported this idea and closely correlated the color of leaves with N concentration [35]. However, studying the variation in nitrogen content and its cause was not within the scope of the current study, and may also be affected by many factors, including the soils of the growing area. Hence, it is apparent that the difference in nitrogen content also affects the growth and other plant components of the accessions and subsequently their biological activities. After the fourth month of transplanting the Moringa accessions to the field, the leaves started to change to yellow and gradually increased from the bottom (older leaves) to upwards (young leaves). However, the current study did not identify the cause of the observed problem, previous studies associated this problem with many factors, such as water stress (either under- or over -watering that leaches nutrients), root damage, excessive levels of organic matter, pest damage, herbicide use, aging of the leaves, high soil pH (alkaline soil) and nutrient deficiencies (iron, zinc, nitrogen, manganese, or phosphorus) [36]. Hence, professional interventions to optimize the growing condition, harvesting stage and nutrient management might be essential to increase production with quality leaves; however, these need to be investigated and confirmed further in future studies. In addition, genetics might be responsible for the difference in adaptability among accessions in the current study; however, it can also be affected by environmental factors such as temperature, light, humidity, water, nutrients and soil characteristics [37,38,39].

All Moringa accession plant extracts of the current study reduced CH_4_ production, which corresponds with several studies that illustrated that Moringa has been one of the most potent medicinal plants to reduce enteric CH_4_ from ruminants [23,24,25,40]. However, it is not possible to be certain of the similarity of accessions between the mentioned previous studies and the current study. The antimethanogenic potential obtained in the current study (18–29%) agreed with the CH_4_ inhibition results of 4.5% (100 mg/L), 5.2% (75 mg/L), 28.7% (50 mg/L) and 29.3% (25 mg/L distilled water) reported using the different dosage levels of *M. oleifera* leaf extracts [40]. However, the lower CH_4_ inhibition potentials of 4.5% (100 mg/L) and 5.2% (75 mL/L) in the abovementioned previous study might be attributed to the difference in the dosage levels of the extract. As stated in various studies, the plant extract bioactivities are highly reliant on the application doses of the metabolites and their thresholds of minimum and maximum activities [41,42,43]. Thus, along with the variation in the type of substrates, mode of application and inclusion levels, the biological differences that exist among the species, varieties and accessions of Moringa also play a significant role in the CH_4_ inhibition obtained in several studies. However, previous studies did not indicate the direct effects and variation of varieties, ecotypes, cultivars, individual plants and plant parts of Moringa on antimethanogenic potential and digestibility, the different antimicrobial activities reported among the mentioned factors [16,44]. In the current study, none of the Moringa accession leaf extracts negatively affected the feed digestion characteristics and kinetics of fermentation. However, the total gas production from anaerobically incubated *E. curvula* hay treated with different Moringa accession extracts was decreased compared with those produced from the control. This was evidenced by their equal or higher OM digestibility when compared with the control, which corresponds to the findings of Dey et al. [45] and Akanmu and Hassen [40]. The lack of significant improvement in OM digestibility and an adverse effect in any of the Moringa accessions implies that Moringa leaf extracts might have stimulatory or no effects on the microbes involved in feed digestibility.

The *M. oleifera* accessions and *M. stenopetala* species of the current study maintained significantly different phytochemical contents of total phenolics and total flavonoids. Most accessions in the current study showed higher total phenolic contents, that range from 3489–4698 mg GAE/100 g extract, compared with previously reported total phenolic contents grown in Chad (2813 mg GAE/100 g), Algeria (3552 mg GAE/100 g) and Haiti (2545 mg GAE/100 g) [16]. However, it was lower than 870–10,870 mg GAE/100 g [15] and 8090–13,644 mg GAE/100 g dried extract [46] reported in Egypt and Southern Tunisia, respectively. Leone et al. [16] associated the variation of polyphenols with their genotypes and environment interaction in the area. However, the genotypic variability of Moringa accessions can be the sole factor responsible for the wide range of phenolic values recorded in the current study. The total flavonoid content in this study also ranged from 1834–2915 mg QE/100 g extract. This yield might be comparable with 1800–2510 mg IQE/100 g [47] total flavonoid contents reported in Thailand. However, it was lower compared with total flavonoid contents of 3170–4420 mg QE/100 g in Southern Tunisia [46], 3120–6280 mg QE/100 g in Egypt [15] and 5570–6026 mg QE/100 g Mexico [48] and higher than 660–1311 mg QE/100 g total flavonoids found in Nigeria [49], whereas the *M. stenopetala* showed 3270 GAE/100 g total phenolic and 1834 QE/100 g total flavonoid contents. These values are lower compared to the total phenolic and total flavonoid contents obtained in most accessions of *M. oleifera* in the current study. However, it was statistically equivalent to the values maintained in *M. oleifera* A1 and A2. Thus, the difference in total phenolic and flavonoid contents obtained in Moringa accessions of the current study indicates the presence of wide ranges of genetic variability among accessions, which play a major role in the variation of phytochemical compositions and contents in Moringa [23]. This could subsequently affect the biological activities of the extracts, including gas production characteristics and antimethanogenic potentials, in different ways. However, the process and accumulation of the PSMs are highly affected by stress and defense response signaling in the plant growing environments, such as extreme temperature, drought, flooding, light, soil fertility and salinity [50,51,52], and their biosynthesis processes are strictly controlled by genes [14,18]. This result is consistent with the findings of previous studies [50,51], and the variations are generally associated with the highest diversification in many characteristics within the *M. oleifera* accessions [23]. 

This study also tried to investigate the relationship between various agronomic performances of the Moringa accessions in the field with in vitro gas production characteristics. Thus, higher CH_4_ inhibitor accessions positively correlated with most agronomic performances (plant height, leaf greenness, plant vigor, canopy diameter and stem diameter) of the respective accessions in the present study. The positive relationships of most agronomic traits of the Moringa accessions with higher CH_4_ inhibition characteristics of their extracts are also supported by another study that found a higher accumulation of PSMs with increased plant height, stem and root diameter as well as biomass yield in woody and perennial medicinal plants [14]. These could ultimately result in better antimethanogenesis during their application in rumen modulation. This may imply that accessions that showed higher adaptability to the growing environment were able to produce higher PSMs in response to that environment, which subsequently increased the bioactivity of the extracts, including different gas production characteristics, antimethanogenesis and OM digestibility. Therefore, this study confirmed that the different agronomic practices that may increase the growth and adaptability of the plants will not negatively affect the efficacy of the extracts on the production characteristics, antimethanogenic potential and digestibility of the feed during their application in rumen modulation and CH_4_ mitigation from ruminants when harvested at a similar physiological stage, as used in the current study. However, measurements and determinations were carried out on 5-month-old leaves, so the results cannot be considered exhaustive for a tree’s life cycle. Thus, additional information on Moringa leaves is necessary to explain the possible sources of variation linked to the season effect (within and among years) and different Moringa plant ages of the same accession. Further study on the relationship of different agronomic practices that increase the growth and yield parameters of the plant, such as fertilizer application, watering frequency and soil characteristics, with antimethanogenesis vs. harvesting stage, also needs to be established in the future.

The antimethanogenic activity of accessions in the current study might also be partly attributed to the total phenolic and total flavonoid contents. This can be evidenced by the recorded positive correlation coefficients of 0.62 and 0.71 with the total phenolic and total flavonoid contents, respectively. However, more information about metabolites and major molecules in the extracts is needed to understand the active ingredients and the way that the molecules interact. Concurrently, *M. oleifera* A3, A8 and A11 contained relatively superior total phenolic and total flavonoid contents and exhibited a higher decrease in CH_4_ production with improved digestibility. However, this relationship was not true for all accessions and the in vitro OM digestibility of incubated feed was not significantly correlated with these phytochemicals in the current study. In agreement with the significant positive correlation coefficients with the CH_4_ inhibition potential of this study, several studies explained that phenolic and flavonoid compounds are the main active components against bacteria, protozoa and fungi [20,21,22]. Similarly, many studies also stated strong antimicrobial activities from metabolites having functional groups of phenolic acids, phenolics and terpenoids [23,24,25,26], which subsequently affects the antimethanogenic potential of the extracts in different ways. The inclusion of some specific flavonoid compounds (flavone, myricetin, naringin, rutin, quercetin, and kaempferol) at 4.5% *w*/*w* DM of the substrate also decreased in vitro CH_4_ production by 5 to 9 mL/g DM, while improved digestibility was obtained only in quercetin and naringin [27]. The mentioned authors associated the antimethanogenic activities of the flavonoids with decreased microbial protein synthesis efficacy and some specific enzymes (filter paperase, xylanase, carboxymethyl cellulase, 𝛽-glucosidase activities, and purine content) [27]. Thus, the effect may vary depending on the structures of the specific flavonoids. This might be part of the reason that all accessions in the current study that recorded higher total phenolic and total flavonoid contents did not show similar CH_4_ inhibition and improvement in OM digestibility of the feed. Therefore, IVOMD is not only dependent on the total phenolic and total flavonoid contents, but also might be affected by other specific metabolites, which agrees with the nonsignificant correlation coefficients of IVOMD with these phytochemicals as revealed by the Pearson correlation coefficients. In addition, the mechanism of increased in vitro digestibility recorded in some of the studied accessions (A1, A2, A3, A8, and A11) may be partly attributed to the antimicrobial and laxative properties reported in Moringa, which makes the rumen environment more conducive for beneficial organisms and enzymes involved in substrate digestion [53]. Based on the findings of this study, four accessions (two higher and two lower CH_4_ inhibitors) were promoted to the next stage of the metabolomics study to identify the candidate metabolite ion-features responsible for their variation in enteric methane inhibition from ruminants [54]. Thus, the published paper [54] is a continuation of this study, which focused on the identification of the most responsible *m/z* ion-features to CH_4_ inhibition characteristics. However, still, the identified metabolite ion-features need to be annotated and investigated for their detailed characteristics (pathway, name, and structure), and the biological effects will be confirmed by using pure compounds in the future.

## 4. Materials and Methods

### 4.1. Plant Growth and Agronomic Data Measurement

Ten *M. oleifera* accessions and one *M. stenopetala* accession were imported from the International Centre for Research in Agroforestry (ICRAF) gene bank in Kenya (Table 7). In addition, the seeds of one *M. oleifera* accession were collected from a private farmer in Pretoria, South Africa. Seedlings of the Moringa accessions were raised in a glasshouse at the University of Pretoria experimental farm. Seeds were sown on seedling trays in the glasshouse on 27 August 2018 and most of them were germinated from 14–28 days after sowing. As suggested by Leone et al. [23], Moringa seedlings can be transplanted within 3–6 weeks after germination. Thus, vigorous seedlings were transplanted to the field at the Roodeplaat experimental site of the Agricultural Research Council (ARC) in the subtropical climate of Pretoria, South Africa, located at 25°44′30′′ S, 28°15′30′′ E on 18 October 2018, i.e., 4–6 weeks after germination. The experimental site was prepared before the transplanting date, i.e., cleaned, fenced, prepared to a fine tilth, and blocked into three that consisted of 12 plots per block with a plot size of 8 m^2^ (2 m × 4 m). Thus, each of the study accessions was randomly placed on a plot in the block and grown in triplicate following a randomized complete block design (RCBD). Each plot was comprised of 15 seedlings in three rows (five seedlings per row) with a 1 m plant distance between rows and within the row. There was a 1.5 m distance between the plots in a block. The plants were irrigated three times a week using a sprinkler and continuously cleaned for the weeds. The seedling survival rate, leaf yield, plant height, canopy diameter, stem diameter, number of primary branches, the score of plant vigor, greenness and chlorosis, and pest and disease incidence traits were recorded for agronomic performance evaluation of the accessions in the study. The data collection techniques of most study parameters were adopted from the International Livestock Research Institute (ILRI) methods applied for the “Evaluation of forage legumes, grasses and fodder trees for use as livestock feed” [55]. The data of agronomic traits were recorded approximately in the fifth month after transplanting to the field, which coincided with the end of the summer season for 2019. 

The percentage of seedling survival rate (% SSR) was calculated from the total number of seedlings transplanted to the field on the transplanting date as follows: $\%\;\mathrm{SSR}=\left(\frac{\mathrm{TNSD}1-\mathrm{NSCT}}{\mathrm{TNSD}1}\right)\times 100,$ where % SSR is the percentage of plant survival rate; TNSD1 is the total number of seedlings transplanted to the field on day 1; and NSCT is the stand count of the plants at five months from the transplanting date. The data of plant height (meter stick), stem diameter (caliper), canopy diameter (meter stick) and primary branches were taken from three to five randomly selected individual plants per plot approximately in the fifth month of transplanting to the field. The score of leaf greenness was given to each plot by comparing the plants with 100% green leaves using a scale of 10 (0 to 9) from light green to deep green, where a score value of 0 was given for plots containing the light green plants while 9 was given for plots containing the highest deep green plants [55]. Similarly, the plant vigor was also assessed by considering how healthy they looked in the field, and each of the plots was scored using a scale of 10 (0 to 9), where a scored value of 0 was given for plots containing the least vigorous plants while 9 was given for plots containing the highest vigorous plants. Furthermore, the accessions were also closely monitored at weekly intervals for signs of pests and disease incidences; however, no disease or pest incidences were observed in the study period.

To determine the leaf yield, all the leaves were harvested approximately in the fifth month of transplanting, except for three to four leaves left to promote the growth of leaves for the next harvest. Two samples per plot or six samples per accession (a total of 72 samples) were collected for the study. The fresh leaves were weighed immediately after harvest and converted into kg of fresh leaf yield per ha. A subsample of the leaf was also taken to determine the moisture content of the leaf to determine kg of leaf DM yield per ha. Thus, the subsample was oven-dried at 105 °C for 24 h until a constant weight was attained, reweighed (DWss) and estimated as follows: $\mathrm{DWL}=\frac{\mathrm{DWSS}}{\mathrm{FWss}}\times \mathrm{FWt}$, where DWL is the dry weight of leaf per plot in g; DWss is the dry weight of the leaf subsample in g; FWss is the fresh weight of the leaf subsample in g and FWt is the total fresh weight of the leaves per plot in g.

### 4.2. Plant Extraction and Determination of Total Phenolic and Total Flavonoid Contents

The harvested leaves were dried using a freeze drier for five days and milled to a 1-mm sieve in a milling machine. Approximately 50 g of the dried leaf powder was suspended in 500 mL methanol at a ratio of 1:10 *w/v* [56] for 96 h in a shaker. The mixture was filtered through a 150 µm aperture sieve (Vickers sieve, Durban, South Africa) and placed in fume chambers for 48 h until semi-dried. The semi-dried crude extracts were further dried using a freeze-drier until a constant weight was attained and kept in the refrigerator at 4 °C until further use. 

One gram of this crude extract was taken from each sample and macerated using 15 mL of 50% methanol to determine the total phenolic content. It was determined following the Folin–Ciocalteu method described by Shah et al. [8] and Madaan et al. [57]. The solution was extracted three times using the same amount of 50% methanol, filtered using Whatman paper, and the volume of the volumetric flask was brought up to 100 mL with 50% methanol. To prepare a gallic acid standard, 25 mg of gallic acid was dissolved in 100 mL of 50% methanol (250 μg/mL) and then further diluted to 12.5, 25, 50, 100, 150 and 200 μg/mL. A 1 mL aliquot of the sample and each dilution of the standard were taken in a separate test tube and diluted with 10 mL of distilled water. Then, 1.5 mL Folin–Ciocalteu reagent was added and incubated at room temperature for 5 min. Four milliliters of 20% (*w*/*w*) Na_2_CO_3_ (sodium carbonate) were added and adjusted with distilled water up to the mark of 25 mL, agitated and left for 30 min at room temperature. Each of the samples and the standard were conducted in triplicate, and their absorbance was measured at 765 nm using a UV/VIS spectrophotometer ANALYTIK JENA AG, Model: SPEKOL 1300 (Germany) against blank distilled water. Eventually, the absorbance readings of the samples in the spectrophotometer were calculated using a standard curve and expressed as mg GAE/g of the extract as follows: $\mathrm{Total}\;\mathrm{phenolic}\;\left(\mathrm{mg}\;\mathrm{GAE}/g\;\right)=\frac{\mathrm{GAE}\times D\times \;V}{W},\;$ where GAE: gallic acid equivalence (mg/mL); D: dilution factor; V: volume of extract (mL); and W: weight of the plant extract (g) used for the preparation of stock solution [8].

The total flavonoid content was also determined using an AlCl_3_ colorimetric assay following the procedures described in Madaan et al. [57] and Bag et al. [58]. Approximately 20 mg of quercetin was dissolved in 100 mL methanol and diluted to 20, 40, 80, 120, 150 and 200 µg/mL using methanol. Aluminum chloride (10%) and 1 M potassium acetate were prepared using distilled water. Then, 0.5 mL of each extract solution and each dilution of the standard were added into a separate graduated test tube in triplicate. In each of the test tubes, 1.5 mL methanol, 0.1 mL aluminum chloride solution, 0.1 mL potassium acetate and 2.8 mL distilled water were added and mixed well. Blanks were also prepared similarly by replacing aluminum chloride solution with distilled water. After 30 min of incubation at room temperature, the absorbance of the mixture was measured at 415 nm against the blank with the specified UV/VIS spectrophotometer. Then, absorbance readings of the samples in the spectrophotometer were calculated and expressed as mg QE/100 g of the extract as follows: $\mathrm{Total}\;\mathrm{flavonoids}\;\left(\mathrm{mg}\;\mathrm{QE}/g\;\right)=\frac{\mathrm{QE}\times D\times V}{W},\;$ where QE: quercetin equivalent obtained from the standard curve (mg/mL); D: dilution factor; V: volume of the extract stock solution (mL); and W: weight of the plant extract (g) [58].

### 4.3. Medium Preparation and In Vitro Incubation

As described by Mould et al. [59], buffer, macromineral and micromineral solutions were prepared before the incubation day and kept in the refrigerator at 4 °C [60]. Incubation for gas measurement was performed with 120 mL serum bottles fitted with modified needle syringe taps that could be opened and closed. Due to the limited capacity of the Inco-shake/incubator, 24 samples coming from each block (two samples per plot) in the field were incubated in triplicate bottles. Thus, a total of 78 bottles, including three control and three blanks, were incubated in each run. Hence, the variation in the rumen fluids used in each incubation and the samples due to the blocks in the field coincided and was accommodated by the RCBD. A previous in vitro study was conducted at four doses (25, 50, 75 and 100 mg extract per kg DM) to determine the dose of certain medicinal plant extracts, including *M. oleifera* and recommended 50 mg extract per kg DM feed for effective antimethanogenic activities [40]. They subsequently used the specified single dose in other studies and obtained good CH_4_ inhibition potentials [61,62], which was also confirmed using our preliminary studies. Thus, the extract was applied at a dose of 50 mg extract per kg DM feed in the study [40]. In order to prepare the recommended dose, well grinded powder of the dried extract was reconstituted with distilled water (5 mg/1000 mL), and 4 mL of the reconstituted extract was added to each 400 mg *E. curvula* hay-containing bottle except for the blanks and the control during incubation. Distilled water, buffer, macromineral and micromineral solutions, tryptone and resazurin solution (0.1%; *w*/*v*) were mixed early in the morning of the incubation day. The mixture was bathed at 39 °C and continuously bubbled with CO_2_ until the incubation process was completed. Rumen fluid was collected from three ruminally cannulated pinzyl (Pinzgauer cross Nguni) steers adapted to *E. curvula* grass hay for approximately 14 days before the start of research. The collection was conducted following the procedures of the approved protocol by the Animal Ethics Committee of the University of Pretoria, South Africa (No. EC039-18). Rumen fluid was collected before the morning feeding of each incubation day. Then, it was transported into the laboratory with a preheated thermoflask under anaerobic conditions within 10–15 min and placed in a water bath set at 39 °C. L-cysteine and Na_2_S.9H_2_O (sodium sulphide nonahydrate) were added to the medium 10 to 15 min before the addition of the rumen fluid. When the solution was sufficiently reduced and changed to colorless, the rumen fluid was sieved with four layers of cheesecloth and mixed with a buffer containing the medium at a 15:25 (*v*/*v*) rumen fluid to medium ratio. Ultimately, 40 mL of the mixture (medium and rumen fluid) was added to all incubation bottles, including the controls and blanks. The bottles were closed with a rubber stopper and transferred into an incubator set at 39 °C with 120 revolutions per minute (rpm). After adding the inoculum to all incubation bottles, the taps were opened for a few seconds to release any built-up gas during the addition process of the inoculum and set a similar starting point. Six runs, two runs per block, were conducted to determine the total gas and CH_4_ production.

In vitro organic matter digestibility (IVOMD) was incubated using two modified digestion phase techniques [63,64]. In the first phase of incubation, 200 mg *E. curvula* grass hay containing extracts of the accessions (50 mg extract/kg DM feed), artificial saliva solution, urea and rumen fluid was incubated under anaerobic conditions for 48 h at 39 °C using a test tube fitted with modified stoppers. In the second phase, an acid and pepsin solution was prepared from 20 mL of 32% HCl and 8 g of pepsin by dissolving them in 2000 mL of distilled water. Then, 20 mL of the prepared acid- and the pepsin-containing solution was added after gently decanting the liquid on the top of the incubation tubes and incubated for another 48 h at 39 °C. After 96 h of the total anaerobic incubation period, the residual plant materials were oven-dried at 100 °C for 18 h and ashed following a standard procedure [63]. In the same way, the incubation was repeated independently three times to determine the IVOMD of *E. curvula* hay treated with extracts of the accessions.

### 4.4. Determination of In Vitro Total Gas, Methane and Organic Matter Digestibility

The total gas was measured with a pressure transducer attached to a digital data logger at 3, 6, 12, 24 and 48 h of incubation [65]. The gas produced during incubation in the bottles was released to the transducer, and the value on the digital data tracker was recorded in pounds’ force per square inch (psi) unit. To obtain the cumulative gas pressure readings of the whole incubation period, each time point reading recorded in psi was added and converted to mL as follows: V*_x_* = V_j_P_psi_ × 0.068004084, where V*_x_* is the gas volume in mL at 39 °C in mL; V_j_ is the headspace of the incubation glass bottle in mL; and P_psi_ is the cumulative pressure recorded by gas monitor system software [66].

Different syringes fitted with stopcocks, which correspond to the incubation bottles, were prepared before the commencement of the research for the collection of CH_4_ samples. The CH_4_ samples were collected at 3, 6, 12, 24 and 48 h, and the CH_4_ concentration in each sample was analyzed by gas chromatography (GC) equipped with a flame ionization detector. It was injected into the GC by a pull and push method, and the area recorded from the GC was converted into parts per million (ppm) using the CH_4_ standard curve. The CH_4_ concentration captured in the headspace was converted to ml by multiplying the total gas produced (mL) by the percent CH_4_ in the sample as follows: CH_4_ (mL) = total gas produced (mL) × % CH_4_ concentration. Blanks were used to correct the total gas and CH_4_ produced by the inoculums. Finally, the total gas and the CH_4_ produced within 48 h of incubation from the substrate feed treated with leaf extracts of the accessions were expressed as mL/g DM incubated feed [44].

As described above, the IVOMD was determined following the two-digestion phase in vitro incubation techniques. Thus, the residual plant materials left after 96 h of total anaerobic incubation were oven-dried at 100 °C for 18 h, measured, and ashed, and the % IVOMD was calculated as $\%\;\mathrm{IVOMD}=\frac{\mathrm{OMFS}-\left(\mathrm{UDR}-\mathrm{Blank}\right)}{\mathrm{OMFS}}\times 100$, where IVOMD: in vitro organic matter digestibility (%); OMFS: the organic matter of the feed sample; UDR: undigested residue.

### 4.5. Statistical Analysis

The RCBD was applied for the statistical analysis of the agronomic traits and in vitro fermentation characteristics to accommodate the variations because of the rumen fluids used in each incubation and sample difference due to the block effect in the field. Before one-way analysis of variance (ANOVA), the data were checked for assumptions of the ANOVA. Significant differences were determined using treatment as a fixed effect: Y_ij_ = μ + α_i_ + β_j_ + ε_ij_, where Yij is the response of treatment i observed in block j; µ is the grand mean; αi is the effect of i^th^ treatment; β_j_ is the effect of the j^th^ block; and εij is the random error. Means were compared using the Tukey test when the F test of a parameter showed a significant difference among accessions *(p* < 0.05). Multivariate principal component analysis (PCA) and hierarchical cluster analysis (HCA) were also carried out to determine the relationship of agronomic traits with the in vitro fermentation characteristics of accessions. SAS version 9.4 [67] and ‘PAST’ free software [68] were used to conduct the statistical analysis in the study.

## 5. Conclusions

The Moringa accessions grown in the subtropical climate of Pretoria, South Africa showed different agronomic performance, total phenolic and total flavonoid contents, methane inhibition potential and organic matter digestibility. Plant extract from *Moringa oleifera* accession 07633 (A3), 07717 (A8) and Pretoria (A11) resulted in higher in vitro methane inhibition with a co-benefit of improved feed organic matter digestibility with equal or higher agronomic performances in the field compared with the other accessions. The positive relationship of total phenolics, total flavonoids and most agronomic traits of the Moringa accessions with methane inhibition potentials obtained in the study improve our knowledge and understanding during the production, standardization, commercialization and utilization of the selected Moringa accession plant extracts as dietary methane mitigation additives. These positive relationships observed in the study suggest that there is a possibility of selecting Moringa accessions for higher methane inhibition potential by selecting for higher plant height, canopy diameter, stem diameter, number of primary branches, plant greenness, plant vigor, leaves yield and seedling survival. However, the results cannot be considered exhaustive for a tree’s life cycle and we require further study on a long-term adaptability performance with concurrent antimethanogenic efficacy to identify the possible sources of variation linked to the season effect (within and among years) and different Moringa plant ages of the same accession, which helps to sustain the efficacy of the plant extract. 

## Figures and Tables

**Table 1:** The mean values of agronomic and growth performances of Moringa accessions at five months of establishment.

Accession Number/Name/Code	PH (cm)	SD (cm)	CD (cm)	NPB	FLY (kg/ha)	DLY (kg/ha)	PVS	PGS	PCS	SS (%)
Bulk (A1)	153.9 ^A^	2.3	72.9 ^AB^	0.53 ^B^	2063.8 ^AB^	553.2 ^AB^	5.33 ^AB^	5.67 ^AB^	2.67	52.5 ^AB^
07229 (A2)	163.6 ^A^	2.8	78.8 ^AB^	1.40 ^AB^	2981.3 ^A^	827.6 ^A^	7.00 ^A^	6.00 ^AB^	2.67	78.1 ^A^
07633 (A3)	188.1 ^A^	3.01	105.3 ^A^	2.67 ^A^	2144.6 ^AB^	608.4 ^AB^	7.67 ^A^	6.33 ^A^	3.67	74.0 ^AB^
07632 (A4)	160.3 ^A^	2.64	95.7 ^AB^	0.80 ^B^	1573.3 ^AB^	470.5 ^AB^	7.00 ^A^	6.33 ^A^	4.00	75.2 ^AB^
07627 (A5)	166.2 ^A^	2.51	82.6 ^AB^	0.20 ^B^	2149.2 ^AB^	589.8 ^AB^	6.33 ^A^	5.00 ^AB^	3.33	74.1 ^AB^
03295 (A6)	127.6 ^AB^	1.89	68.2 ^AB^	0.68 ^B^	590.4 ^B^	168.2 ^B^	5.67 ^AB^	5.33 ^AB^	2.67	61.9 ^AB^
05536 (A7)	125.9 ^AB^	1.95	69.1 ^AB^	0.47 ^B^	1458.3 ^AB^	423.0 ^AB^	4.67 ^AB^	5.33 ^AB^	3.33	51.9 ^AB^
07717 (A8)	150.8 ^A^	2.47	74.5 ^AB^	0.73 ^B^	2107.5 ^AB^	604.1 ^AB^	7.00 ^A^	6.33 ^A^	3.33	75.4 ^AB^
07316 (A9)	138.3 ^AB^	2.14	74.3 ^AB^	1.07 ^B^	1697.9 ^AB^	468.2 ^AB^	5.67 ^AB^	6.33 ^A^	4.00	58.2 ^BC^
07216 (A10)	105.1 ^AB^	1.59	58.9 ^B^	0.27 ^B^	805.0 ^B^	220.2 ^B^	4.33 ^AB^	5.67 ^AB^	3.00	44.9 ^B^
Pretoria (A11)	146.7 ^A^	2.12	69.1 ^AB^	0.87 ^B^	1975.4 ^AB^	552.4 ^AB^	6.00 ^AB^	5.67 ^AB^	4.00	73.7 ^AB^
*M. stenopetala* (MS)	46.7 ^B^	1.67	60.0 ^B^	1.27 ^AB^	1452.9 ^AB^	421.2 ^AB^	2.00 ^B^	4.33 ^B^	1.67	59.2 ^AB^
*p* value	0.002	0.073	0.009	0.001	0.006	0.009	0.006	0.027	0.328	0.0053

Within a column mean values with different superscript letters significantly different at *p* < 0.05. PH: plant height; SD: stem diameter; CD: canopy diameter; NPB: number of primary branches; FLY: fresh leaf yield; DLY: dry leaf yield; PVS: plant vigor score; PGS: plant greenness score; PCS: plant chlorosis score; SS: seedling survival rate (%).

**Table 2:** The principal component loadings of the agronomic traits of the Moringa accessions recorded at five months of the establishment year.

Agronomic Traits	PC 1 × 100	PC 2 × 100	PC 3 × 100	PC 4 × 100
Canopy diameter	33.88	−7.02	16.75	12.44
Chlorosis score	23.90	−30.53	−41.19	10.59
Dry leaf yield	24.45	49.67	−1.67	−53.63
Fresh leaf weight	23.70	50.57	−3.87	−6.37
Number of primary branches	20.78	2.88	66.56	−5.15
Plant greenness score	27.69	−16.65	−29.44	−12.39
Plant height	33.72	−1.41	−27.96	43.72
Plant vigor score	34.81	−4.44	−23.9	−8.61
Stem diameter	35.42	17.61	4.44	62.66
Seedling survival rate	28.35	21.87	7.18	3.73
Total flavonoids	26.47	−40.42	24.97	1.93
Total phenolics	28.56	−35.58	25.70	−26.59
Eigenvalue	7.09	2.14	1.21	0.58
% variance	59.09	17.8	10.07	4.81

PC: principal component.

**Table 3:** The in vitro fermentation characteristics of *Eragrostis curvula* hay incubated with leaf extracts from Moringa accessions (Mean ± SEM).

Accession Number/Code	Mean Value ± SEM				
	TGP (mL/g DM)	CH_4_ (mL/g DM)	CH_4_/IVOMD (%)	IVOMD (%)	CH_4_ Inhibition (%)
Bulk (A1)	170.5 ± 1.2 ^BCD^	5.23 ± 0.13 ^B^	8.81 ± 0.18 ^BCD^	59.3 ± 0.4 ^A^	17.9 ± 2.0 ^B^
07229 (A2)	172.7 ± 1.6 ^B^	5.21 ± 0.17 ^B^	8.70 ± 0.36 ^DE^	60.0 ± 0.9 ^A^	18.2 ± 2.0 ^B^
07633 (A3)	160.8 ± 2.2 ^E^	4.56 ± 0.15 ^C^	7.61 ± 0.37 ^F^	60.03 ± 1.0 ^A^	28.4 ± 2.1 ^A^
07632 (A4)	164.4 ± 0.5 ^CDE^	4.71 ± 0.14 ^C^	8.69 ± 0.21 ^DE^	54.2 ± 0.7 ^BC^	25.9 ± 2.0 ^A^
07627 (A5)	163.7 ± 2.8 ^DE^	4.72 ± 0.17 ^C^	8.79 ± 0.18 ^CD^	53.7 ± 0.9 ^C^	26.0 ± 2.3 ^A^
03295 (A6)	169.9 ± 3.4 ^BCD^	5.06 ± 0.23 ^B^	9.41 ± 0.35 ^BCD^	53.8 ± 0.5 ^C^	20.6 ± 3.4 ^B^
05536 (A7)	170.4 ± 2.7 ^BCD^	5.04 ± 0.18 ^B^	9.38 ± 0.36 ^BC^	53.7 ± 0.9 ^C^	20.9 ± 2.7 ^B^
07717 (A8)	164.2 ± 1.6 ^CDE^	4.68 ± 0.16 ^C^	8.06 ± 0.21 ^EF^	58.4 ± 0.5 ^A^	26.6 ± 2.3 ^A^
07316 (A9)	170.6 ± 1.9 ^BC^	5.01 ± 0.13 ^B^	9.22 ± 0.20 ^BCD^	54.3 ± 1.1 ^BC^	21.4 ± 2.0 ^B^
07216 (A10)	170.2 ± 1.6 ^BCD^	5.04 ± 0.18 ^B^	9.22 ± 0.35 ^BCD^	54.7 ± 1.3 ^BC^	20.9 ± 2.8 ^B^
Pretoria (A11)	164.1 ± 0.4 ^CDE^	4.52 ± 0.18 ^C^	7.85 ± 0.28 ^F^	57.6 ± 1.0 ^A^	29.1 ± 2.6 ^A^
*M. stenopetala* (MS)	175.0 ± 1.1 ^B^	5.17 ± 0.15 ^B^	9.45 ± 0.24 ^B^	54.7 ± 0.9 ^BC^	18.8 ± 2.1 ^B^
Control	203.8 ± 5.3 ^A^	6.33 ± 0.18 ^A^	11.65 ± 0.40 ^A^	54.7 ± 0.4 ^BC^	-

Within a column means with different superscript letters significantly (*p* < 0.01) different. TGP: total gas produced; IVOMD: in vitro organic matter digestibility; SEM: standard error of the mean.

**Table 4:** The total phenolic and total flavonoid contents of Moringa accession extracts (mean ± SEM) and their Pearson correlation coefficients with major in vitro fermentation characteristics.

Accessions Number/Name	Total Phenolic (mg GAE/100 g Extract)	Total Flavonoid (mg QE/100 g Extract)
Bulk (A1)	3510 ± 83 ^CD^	1834 ± 70 ^D^
07229 (A2)	3489 ± 258 ^CD^	1894 ± 168 ^CD^
07633 (A3)	4698 ± 75 ^A^	2915 ± 68 ^A^
07632 (A4)	3927 ± 71 ^BC^	2391 ± 42 ^B^
07627 (A5)	3671 ± 122 ^BCD^	2311 ± 120 ^B^
03295 (A6)	3661 ± 90 ^BCD^	2159 ± 84 ^BCD^
05536 (A7)	3586 ± 157 ^BCD^	2102 ± 43 ^BCD^
07717 (A8)	3998 ± 93 ^B^	2250 ± 126 ^B^
07316 (A9)	3578 ± 7 ^BCD^	2083 ± 78 ^BCD^
07216 (A10)	3770 ± 101 ^BC^	2190 ± 149 ^BC^
Pretoria (A11)	3800 ± 89 ^BC^	2275 ± 105 ^B^
*M. stenopetala* (MS)	3270 ± 181 ^D^	1834 ± 144 ^D^
Pearson correlation coefficients (*2-tailed*)		
Total gas	−0.70551 *	−0.73034 *
CH_4_	−0.62109 *	−0.70695 *
% CH_4_ inhibition	0.62057 *	0.70699 *
IVOMD	0.32318	0.1105
CH_4_/IVOMD	−0.67362 *	−0.61938 *

Within a column means of accessions with different superscript letters significantly different at *p* < 0.01. The Pearson correlation coefficients with superscript * indicate the significant correlation between the parameters at *p* < 0.01. IVOMD: in vitro organic matter digestibility; QE: quercetin equivalent; GAE: gallic acid equivalent.

**Table 5:** Correlation of agronomic traits of Moringa accessions with total phenolic and total flavonoid contents and in vitro fermentation characteristics of *Eragrostis curvula* hay treated with their extracts (Pearson correlation, 2-tailed).

Agronomic Traits	Metabolites		In Vitro Fermentation Characteristics					
	TP	TF	TGP	CH_4_	IVOMD	CH_4_/IVOMD	CH_4_/TGP	% CH_4_ Inhibition
CD	0.452 **	0.438 **	−0.475 **	−0.307	0.203	−0.366 *	−0.143	0.308
DLY	0.044	−0.065	−0.206	−0.143	0.424 **	−0.369 *	−0.09	0.143
FLY	0.027	−0.08	−0.178	−0.105	0.452 **	−0.356 *	−0.054	0.105
NPB	0.421 *	0.375 *	−0.243	−0.249	0.404 *	−0.422 **	−0.226	0.25
PCS	0.203	0.246	−0.369 *	−0.244	−0.007	−0.194	−0.166	0.246
PGS	0.360 *	0.258	−0.426 **	−0.428 **	0.219	−0.470 **	−0.361 *	0.428 **
PH	0.430 **	0.363 *	−0.476 **	−0.299	0.361 *	−0.459 **	−0.161	0.3
PVS	0.453 **	0.425 **	−0.464 **	−0.311	0.318	−0.441 **	−0.188	0.312
SD	0.332 *	0.261	−0.372 *	−0.242	0.356 *	−0.408 *	−0.143	0.243
SS	0.294	0.291	−0.367 *	−0.359 *	0.273	−0.455 **	−0.318	0.359 *

The Pearson correlation coefficients with superscript ** indicate a significant correlation between the parameters at *p* < 0.01, whereas * shows a significant correlation at *p* < 0.05. CD: canopy diameter; DLY: dry leaf yield; FLY: fresh leaf yield; NPB: number of primary branches; PCS: plant chlorosis score; PGS: plant greenness score; PH: plant height; PVS: plant vigor score; SD: stem diameter; SS: seedling survival rate; TP: Total phenolics; TF: total flavonoids; TGP: total gas production; IVOMD: in vitro organic matter digestibility.

**Table 6:** Principal component loadings of the agronomic traits of the Moringa accessions and in vitro fermentation characteristics of *Eragrostis curvula* hay treated with their extracts.

Parameters	PC 1 × 100	PC 2 × 100	PC 3 × 100	PC 4 × 100
Canopy diameter (CD)	26.21	6.86	20.92	13.74
CH_4_	−25.63	27.39	22.32	7.11
% CH_4_ inhibition	25.56	−27.61	−22.02	−7.75
CH_4_/IVOMD	−28.13	−2.99	21.77	−7.59
CH_4_/TGP	−20.67	29.93	38.51	7.92
Chlorosis score (ChS)	21.69	−21.17	24.67	−31.23
Dry leaf yield (DLY)	17.51	39.91	−23.20	−18.36
Fresh leaf yield (FLY)	16.78	40.97	−22.00	−19.51
IVOMD	15.82	35.77	−9.44	24.32
Number of primary branches (NPB)	15.88	15.81	−8.19	64.02
Plant greenness score (PGS)	21.16	5.91	45.18	−5.15
Plant height (PH)	26.16	11.54	29.78	−20.72
Plant vigor score (PVS)	27.48	8.47	26.10	−17.71
Stem diameter (SD)	26.44	24.96	9.95	−1.81
Seedling survival (SS)	23.75	12.15	−29.17	−16.33
Total flavonoids (TF)	25.00	−24.89	8.57	28.03
Total gas production (TGP)	−27.75	20.75	3.01	6.55
Total phenolics (TP)	26.02	−15.05	13.89	36.48
Eigenvalue	10.26	3.27	1.42	1.27
% variance	57.00	18.19	7.89	7.08

PC: principal component.

**Table 7:** The germplasm of Moringa accessions, which were imported from the International Centre for Research in Agroforestry in Kenya and collected in Pretoria, South Africa.

Species Name	Accession Code/Number	Collection Area	Country of Origin
*Moringa oleifera*	A1 (Bulk)	Meru	Kenya
*Moringa oleifera*	A2 (7229)	Machakos	Kenya
*Moringa oleifera*	A3 (7633)	Segou	Mali
*Moringa oleifera*	A4 (7632)	Bamako	Mali
*Moringa oleifera*	A5 (7627)	NA	Kenya
*Moringa oleifera*	A6 (3295)	Mbbololo	Kenya
*Moringa oleifera*	A7 (5536)	Busia	Kenya
*Moringa oleifera*	A8 (7717)	Ramogi	Kenya
*Moringa oleifera*	A9 (7316)	Kibwezl	Kenya
*Moringa oleifera*	A10 (7216)	Ramisi	Kenya
*Moringa oleifera*	A11 (NA)	Pretoria	South Africa
*Moringa stenopetala*	MS (NA)	NA	Kenya

NA: not available.

## Data Availability

The study data will be deposited in the University of Pretoria repository. Thus, it will be accessed via requesting the corresponding author or the University of Pretoria.
